# Exercise Promotion and Distress Reduction Using a Mobile App-Based Community in Breast Cancer Survivors

**DOI:** 10.3389/fonc.2019.01505

**Published:** 2020-01-10

**Authors:** Il Yong Chung, Miyeon Jung, Yu Rang Park, Daegon Cho, Haekwon Chung, Yul Ha Min, Hye Jin Park, Minsun Lee, Sae Byul Lee, Seockhoon Chung, Byung Ho Son, Sei-Hyun Ahn, Jong Won Lee

**Affiliations:** ^1^Department of Surgery, Asan Medical Center, University of Ulsan College of Medicine, Seoul, South Korea; ^2^Korea Advanced Institute of Science and Technology, Seoul, South Korea; ^3^Department of Biomedical Systems Informatics, Yonsei University College of Medicine, Seoul, South Korea; ^4^Swallaby Co., Ltd., Seoul, South Korea; ^5^College of Nursing, Gachon University, Incheon, South Korea; ^6^Department of Psychiatry, Asan Medical Center, University of Ulsan College of Medicine, Seoul, South Korea

**Keywords:** telemedicine, breast neoplasms, mobile applications, quality of life, psychological stress, exercise, smartphone, survivorship

## Abstract

Physical activity (PA) enhancement and mental distress reduction are important issues in cancer survivorship care. Mobile technology, as an emerging method for changing health behaviors, is gaining attention from many researchers. This study aimed to investigate the effect of a mobile app-based community on enhancing PA and decreasing distress in breast cancer survivors. We conducted a non-randomized, prospective, interventional study that had a mobile community-later arm and mobile community-first arm. With an Android smartphone app (WalkON®), daily walk steps and weekly distress scores using app-based Distress Thermometer (DT) questionnaires were collected from participants for about 12 weeks. To examine the difference in weekly step counts before and during the community activity, we used a paired *t*-test method. For a comparative analysis, we referred to a previous prospective observational study without a mobile community intervention that had the same setting as the present study. After propensity score matching (PSM), multivariable regression modeling with difference-in-difference (DID) was performed to estimate the effect of the mobile app-based community on PA and mental distress. From January to August 2018, a total of 64 participants were enrolled in this study. In the univariate analysis, after participation in the mobile community, the participants showed a significant increase in total weekly steps (*t* = −3.5341; *P* = 0.00208). The mean of the differences was 10,408.72 steps. In the multivariate analysis after PSM, the mobile community significantly increased steps by 8,683.4 per week (*p* value <0.0001) and decreased DT scores by 0.77 per week (*p* value = 0.009) in the mixed effect model. In the two-way fixed effect model, the mobile community showed a significant increase in weekly steps by 8,723.4 (*p* value <0.0001) and decrease in weekly DT by 0.73 (*p* value = 0.013). The mobile app-based community is an effective and less resource-intensive tool to increase PA and decrease distress in breast cancer survivors.

**Trial Registration:** NCT03190720, NCT03072966

## Introduction

Many cancer survivors experience psychological distress after cancer diagnosis. Depression and anxiety have a prevalence of 11.6 and 17.9%, respectively, among cancer survivors (1). A systematic review reported 22 and 10%, respectively, as the prevalence of depression and anxiety specifically among breast cancer survivors (2). The National Comprehensive Cancer Network (NCCN) guidelines recommend that cancer patients should be managed for their distress regularly according to clinical practice guidelines (3).

Pharmacologic and/or non-pharmacologic interventions can be used to manage depressive symptoms in cancer survivors (4). Exercise is one of the important non-pharmacologic interventions for mental distress in breast cancer survivors. Many breast cancer survivors are not physically active, which is generally associated with poorer health-related quality of life (HRQOL) (5). Interventions to enhance physical activity (PA) may improve mental HRQOL in breast cancer survivors who have completed cancer therapy. Moreover, PA can increase overall survival in breast cancer survivors and decrease treatment-related adverse effects, such as fatigue, thereby leading to better QOL. The Breast Cancer Survivorship Care Guidelines from the American Cancer Society and American Society of Clinical Oncology recommend that breast cancer survivors avoid inactivity and do at least 150 min of moderate or 75 min of vigorous aerobic exercise every week (6).

To enhance PA in cancer survivors, a number of psychological theories related to behavioral change and methodologies have been applied (6). Studies to increase PA in breast cancer survivors have shown that interventions such as direct interviews or regular telephone calls to participants could significantly increase PA levels. However, these interventions are so resource-intensive that clinicians cannot easily perform them in real-world daily practice (7, 8).

Recently, mobile technology, as an emerging method for changing health behaviors, is gaining academic attention (9). Mobile app-based health promotion programs can monitor users' health status and provide health information and feedback, which may lead to behavior change. However, most previous studies have been conducted on general populations, not in cancer survivors. Moreover, although these mobile app-based programs increase PA in cancer survivors, their distress-reducing effects, by increasing PA, have not been investigated.

The present study aimed to investigate the effects of a mobile app-based community program on enhancing PA, and assess whether the mobile community program decreases mental distress through increasing PA in breast cancer survivors.

## Methods

### Participants

We conducted a non-randomized, prospective, interventional study (NCT03190720) enrolling female patients who were hospitalized for breast cancer surgery at Asan Medical Center. Patients were included if their ages were between 20 and 60 years and their own Android smartphone was compatible with the free activity-tracking app modified for this study. Patients were excluded if they had metastatic or recurrent breast cancer, or were not capable of using a smartphone. Patients who were pregnant, resided in a foreign country, or were scheduled for adjuvant chemotherapy were also excluded. After enrollment, if adjuvant chemotherapy was decided according to final pathologic reports, these patients were also excluded. Participants were assigned to two arms: (1) mobile community-later arm, in which participants were followed without being registered to a mobile community at first and then registered to the mobile community after about 6 weeks; (2) mobile community-first arm, in which participants were registered to the abovementioned mobile community at first and then dropped from the mobile community and followed after 6 weeks.

We also included a previous prospective study for a comparative analysis (NCT03072966). The previous study was conducted to develop distress-screening algorithms using mobile devices in breast cancer survivors who had never been registered to a mobile community (10), and otherwise, the platform of study design was similar to that of the present study. We used this group of patients as a control group who did not participate in the mobile community.

Written informed consent was obtained from all participants at study enrollment. The study protocol was approved by the institutional review board at Asan Medical Center (2017-0328). These studies were registered on the ClinicalTrial.gov website (NCT03190720, NCT03072966).

### Study Setting

During the hospital stay after breast cancer surgery, patients were contacted by a clinical research assistant. After consenting to participate, the participants completed paper-based questionnaires (Distress Thermometer, DT) at baseline. The Android-based app for this study was downloaded to the participants' smartphones.

At the start of the study, patients were allocated to the mobile community-later group. After the 20th participant was followed for 4 weeks, we opened a mobile app-based community and registered 20 participants at the same time. Originally, this study was designed to register participants in the mobile community-later group to the mobile community 6 weeks after enrollment. However, if the first participant goes into the mobile community 6 weeks after enrollment, then this participant would be alone, which means this participant is not in an actual community. Thus, we gathered 20 participants and then opened the mobile community.

### Mobile App for Data Collection and Mobile Community

We used a free mobile health care app (WalkOn®, Swallaby Co., Ltd., Seoul, Republic of Korea, http://www.swallaby.com/) as a study platform. This app (10) was modified to obtain users' daily step count and app-based questionnaires on weekly DT (3). The participants were instructed to open and pull-to-refresh the app at least once a week to send the daily walking data to a central database system that archives the anonymized data for each participant. The participants were also asked to report DT once a week.

In this app, we opened the mobile app-based community for an intervention where users can view other members' daily step count to motivate them and promote health-related activities. Health information on diet and PA was posted on the board in the mobile app-based community once a week.

### Statistical Analysis

The balance among the participant demographics, clinicopathological, and intervention variables was compared using a chi-squared test or univariate analysis of variance (ANOVA) according to assigned group.

To examine the effect of the mobile app-based community on weekly step count before and during community activity in the mobile community-later group, we performed a paired *t*-test. To test the persistence of the mobile community effect, we also tested the difference in step counts during and after community activity using a subsample of the community-first group.

Multivariable regression modeling with difference-in-difference (DID) was performed to estimate the effect of the mobile app-based community on PA and mental distress between the control and community-later groups. To reduce the effect of selection bias in the quasi-experimental studies, we employed both mixed and two-way fixed effects models. Dependent variables were the weekly total steps and weekly average scores of DT.

We employed standard DID method to analyze the effect of the mobile community on PA. Regressions included an indicator variable for the time period of PA measurement (i.e., pre- vs. post-mobile app-based community initiation), an indicator variable for community group (control vs. mobile community-later), and the interaction of these two indicators. The indicator for time period corresponded to the difference in steps for participants before vs. after community initiation. The indicator for control and community-later groups corresponded to the difference between the two groups at baseline. The interaction term reflected the DID parameter (i.e., change in steps with the intervention of app-based community relative to change for the control group).

Individual participants (fixed effect) and months (random effect) were included as control variables in a linear mixed-effects model. In the two-way fixed-effects model, the week (fixed effect) was included as control variable instead of the month.

1. Mixed effects model (monthly random effects + individual fixed effects)
  $$\begin{array}{l} y_{it}=\beta_{1}{During\_Community}_{it}+\beta_{2}{Community\_later}_{i}\\ \;\times {During\_Community}_{it}+\;{Month}_{t}+\;{Patient\_Fixed}_{i}+\epsilon_{it} \end{array}$$
2. Two-way fixed effects model (weekly fixed effects + individual fixed effects)
  $$\begin{array}{l} y_{it}=\beta_{3}{{Community\_later}_{i}\times During\_Community}_{it}\\ \;+\;{Weekly\_Fixed}_{t}+\;{Patient\_Fixed}_{i}+\epsilon_{it} \end{array}$$

As the purpose of this analysis was to estimate the effect of the community compared with the control group, without the confounding effect of the time trend, we used a panel of two groups (except the community-first group). The panel data were constructed using the data before and after 6 weeks from the initiation of the community intervention.

In addition, to decrease the risk of biased estimates of the intervention effect, we defined the logit of predicted probability of intervention as a propensity score using the following patient characteristics: age, marital status, education level, employment status, comorbidity, episode of depression, anti-hormonotherapy, and chemotherapy. The balance check was performed again after matching the control and community-later groups using propensity score matching (PSM). Matching was carried out using a ratio of 1:2, and a caliper distance of 0.025, without replacement based on nearest-neighbor matching. All analyses were conducted using R Software (version 3.6.1, R Foundation for Statistical Computing, Vienna, Austria), and a *p* value of <0.05 was considered to indicate statistical significance.

## Results

### Descriptive Statistics

**Figure 1:**
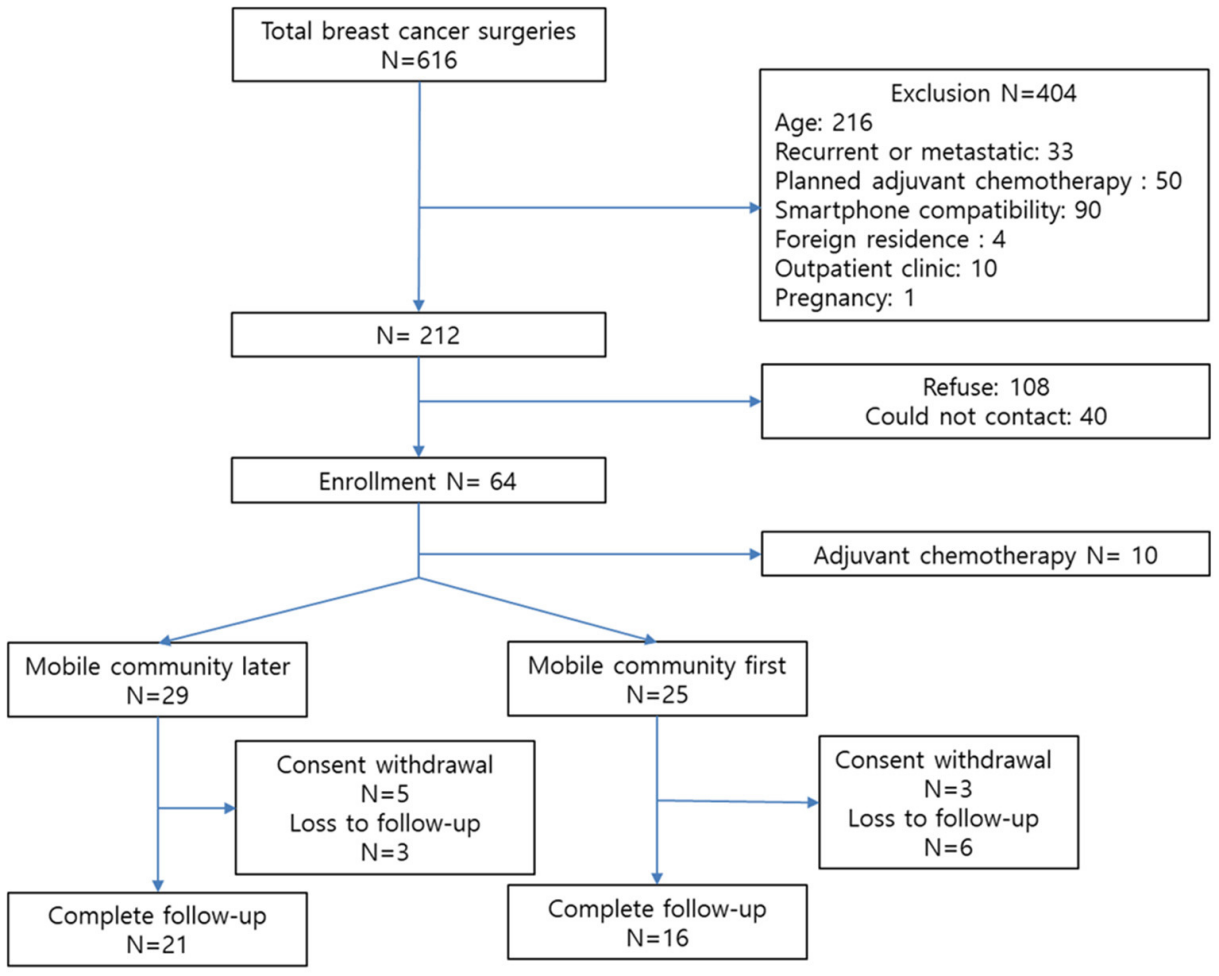
Study enrollment.

From January to August 2018, a total of 64 participants were enrolled (Figure 1). Following enrollment, 10 patients whose adjuvant treatment plans were changed according to postoperative pathologic results were excluded. After 3 months of follow-up, the number of participants in the mobile community-later and -first groups were 21 and 16, respectively. We included 160 patients from the previous prospective study for the comparative analysis (10).

Table 1 summarizes the descriptive statistics of the demographics and clinical characteristics of the participants as absolute and relative frequencies. The mean age of patients was 44.45 (SD 6.42) years. Most patients were married (82.50%) and had completed post-secondary education (71.88%). Of all, 58 patients (36.25%) were employed full-time. A total of 106 patients (66.25) were diagnosed with comorbidities. Only one patient (0.63%) was stratified into the group that experienced an episode of depression; 59 patients (36.88%) were stratified into the group that received chemotherapy; and 134 patients (83.75%) received anti-hormonal therapy.

**Table 1:** Baseline characteristics.

	**Full sample**	**Mobile community-later**	**Mobile community-first**	**Control**	***P* value**
*n*	160	21	16	123	
Age at diagnosis, years (std)	44.45 (6.418)	43.762 (5.770)	40.313 (5.896)	45.106 (6.418)	0.016
Married, *n* (%)	132 (82.500)	12 (57.143)	15 (93.75)	105 (85.366)	0.015
Bachelor's degree, *n* (%)	115 (71.875)	17 (80.952)	12 (75)	86 (69.919)	0.564
Employed, *n* (%)	58 (36.25)	7 (33.333)	9 (56.25)	42 (34.146)	0.214
Comorbidity, *n* (%)	106 (66.25)	10 (47.619)	8 (50)	88 (71.545)	0.035
Episode of depression, *n* (%)	1 (0.625)	1 (4.762)	0 (0)	0 (0)	0.400
Previous chemotherapy, *n* (%)	59 (36.875)	4 (19.048)	3 (18.75)	52 (42.276)	0.036
Anti-hormonal therapy, *n* (%)	134 (83.75)	16 (76.190)	13 (81.25)	105 (85.366)	0.490

*std, standard deviation*.

No difference was seen among patients who were in the control, mobile community-later, and community-first groups in terms of education level, employment, episode of depression, and anti-hormonal therapy. However, age at diagnosis, marital status, comorbidity, and previous chemotherapy were different depending on group.

### Improvement of Weekly Step Outcome

**Figure 2:**
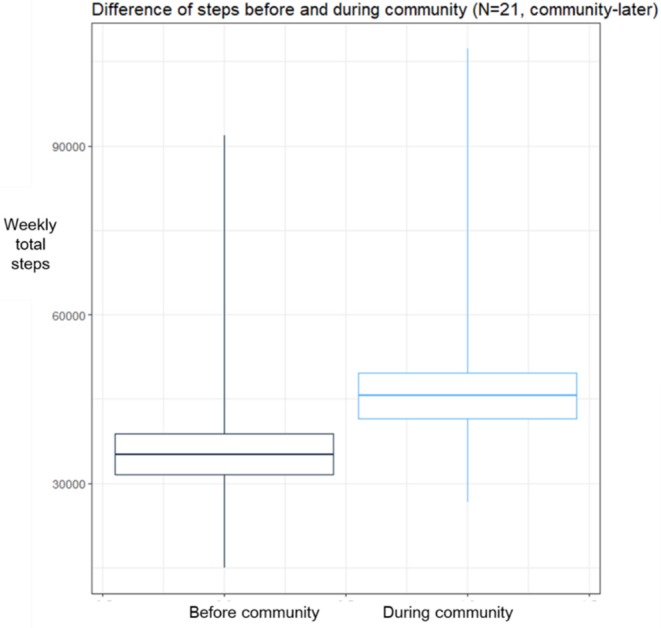
Comparison of weekly step counts. Boxplot means ±1*standard deviation of sample. After-intervention significantly higher than before-intervention period.

Figure 2 shows a comparison of weekly total step counts before and during community activity in the mobile community-later group. After participation in the mobile community, the participants showed a significant increase in total weekly steps (*t* = −3.5341; *P* = 0.00208). The mean of the differences was 10,408.72 steps.

In the mobile community-first group, the weekly total steps seemed to decrease, but the difference was not statistically significant (*t* = 0.98896; *P* = 0.3384) according to a paired *t*-test (Supplementary Figure 1). The mean of the differences was 3,869.103 steps.

### DID With Control and Mobile Community-Later Groups Before PSM

Table 2, panel A lists the results from the multivariate regression analysis for weekly total step measure. Controlling for time and individual-idiosyncratic effects, we found that patients treated at the community in the later (post-community initiation) time period were significantly more likely to walk compared with patients treated at the control group in both mixed and fixed effects models.

**Table 2:** Effect of mobile app-based community on weekly steps and mental distress (before PSM).

**Panel A: Weekly total steps**				
	**Mixed effects model**		**Two-way fixed effects model**	
	**B (SE)**	***P*** **value**	**B (SE)**	***P*** **value**
Time 1 (vs. time 0)^†^	4763.3 (1273.8)	0.0002	NA	NA
Community-later group change in steps between times 0 and 1 (vs. control group change)^§^	6116.3 (1705.1)	0.0003	6177.2 (1697.7)	0.0003
Number of patients	144		144	
Observation (number of weeks * number of patients)	1,770		1,770	
R-squared	0.6852		0.6895	
**Panel B: Weekly average DT**				
	**Mixed effects model**		**Two-way fixed effects model**	
	**B (SE)**	***P*** **value**	**B (SE)**	***P*** **value**
Time 1 (vs. time 0)^†^	0.2918 (0.1926)	0.1303	NA	NA
Community-later group change in DT score between times 0 and 1 (vs. control group change)^§^	−0.4340 (0.2035)	0.0334	−0.4355 (0.2026)	0.0321
Number of patients	99		99	
Observation (number of weeks * number of patients)	651		651	
R-squared	0.8047		0.8095	

*PSM, propensity score matching; SE, standard error; DT, Distress Thermometer; NA, not available*.

† *Time variable*,

§ *Difference-in-difference term, examining difference over time between community-later and control groups*.

Compared with the simple before-and-after comparison for the community-later group using paired *t*-test in Figure 2, this DID analysis could define the natural time trend of weather change. As shown in the coefficient of Time 1, in the post-community initiation period, the weekly steps for patients were significantly greater by as much as 4,763 steps. Therefore, compared with the increase of 10,000 steps in paired *t*-test, the effect of community in DID analysis was about 6,100–6,200 steps after removing the time trend effect of about 4,500 steps.

We then tested the difference in weekly average DT scores, the second measure of community effect. Table 2, panel B lists the results from the multivariate regression analysis for weekly average DT scores. Controlling for time and individual-idiosyncratic effects, we found that patients treated at the community in the later (post-community initiation) time period had significantly lower DT scores compared with patients treated at the control group in both mixed and fixed effects models by as much as 0.4 points.

### DID With a Matched Control Group: Weekly Steps and Distress Level

A total of 123 control and 21 community-later patients were eligible for matching. The matching process resulted in a final cohort of 63 patients (42 control and 21 community-later patients) eligible for further analysis. The patient characteristics are summarized in Table 3. The mean ages of the control and community-later cohorts were 43.7 and 43.0 years, respectively. Comorbidities were reported by 47.6 and 64.2% in the control and community-later cohorts, respectively. All differences in covariates were statistically insignificant (*P* > 0.1).

**Table 3:** Characteristics of all propensity-matched patients.

	**Full sample**	**Community-later**	**Control**	***P* value**
*n*	63	21	42	
Age at diagnosis, years (std)	43.3016 (5.3420)	43.7619 (5.7698)	43.0714 (5.1721)	0.6460
Married, *n* (%)	45 (71.4286)	12 (57.1429)	33 (78.5714)	0.0952
Bachelor's degree, *n* (%)	49 (77.7778)	17 (80.9524)	32 (76.1905)	0.7573
Employed, *n* (%)	25 (39.6825)	7 (33.3333)	18 (42.8571)	0.6489
Comorbidity, *n* (%)	37 (58.7302)	10 (47.6191)	27 (64.2857)	0.3196
Episode of depression, *n* (%)	1 (1.5873)	1 (4.7619)	0 (0)	0.3333
Chemotherapy, *n* (%)	8 (12.6984)	4 (19.0476)	4 (9.5238)	0.4234
Anti-hormonal therapy, *n* (%)	49 (77.7778)	15 (76.1905)	33 (78.5714)	1

*std, standard deviation*.

For both measures, the differences were statistically significant; our previous results were robust toward the PSM. Table 4, panel A lists the results from the multivariate regression analysis for weekly total steps, and Table 4, panel B, the results of the effect of community on weekly average DT scores. Controlling for time and individual-idiosyncratic effects, the analyses showed that patients treated at the community in the later (post-community initiation) time period were significantly more likely to walk compared with patients treated in the control group in both mixed and fixed effects models by as much as about 8,700 steps.

**Table 4:** Effect of mobile app-based community on weekly steps and mental distress after PSM.

**Panel A: Weekly total steps**				
	**Mixed effects model**		**Two-way fixed effects model**	
	**B (SE)**	***P*** **value**	**B (SE)**	***P*** **value**
Time 1 (vs. time 0)^†^	2128.47 (2053.72)	0.3	NA	NA
Community-later group change in steps between times 0 and 1 (vs. control group change)^§^	8683.40 (1992.87)	0.00001	8723.35 (1977.94)	0.00001
Number of patients	63		63	
Observation (number of weeks * number of patients)	772		772	
R-squared	0.5957		0.6065	
**Panel B: Weekly average DT**				
	**Mixed effect model**		**Two-way Fixed effect model**	
	**B (SE)**	**P value**	**B (SE)**	**P value**
Time 1 (vs. time 0)^†^	0.4537 (0.3384)	0.1812	NA	NA
Community-later group change in DT score between times 0 and 1 (vs. control group change)^§^	−0.7724 (0.2933)	0.009	−0.7328 (0.2916)	0.0126
Number of patients	46		46	
Observation (number of weeks * number of patients)	308		308	
R-squared	0.7606		0.7729	

*PSM, propensity score matching; SE, standard error; DT, Distress Thermometer; NA, not available*.

† *Time variable*,

§ *Difference-in-difference term, examining difference in steps over time between community-later and control groups*.

In terms of DT, controlling for time and individual-idiosyncratic effects, patients treated at the community in the later (post-community initiation) time period had significantly lower DT scores compared with patients treated in the control group in both mixed and fixed effects models by as much as about 0.8 points.

## Discussion and Conclusion

Our results indicated that the selected mobile app-based community was effective to increase PA and decrease mental distress in breast cancer survivors. Breast cancer survivors after breast cancer surgery who participated in the mobile community walked significantly more steps per week, by around 7,500 steps, compared with counterparts who did not register in the mobile community. Further, the level of mental distress estimated by DT was significantly lowered in breast cancer survivors in the mobile community compared with the non-community group.

To our knowledge, this prospective study is the first to investigate the effect of mobile app-based community on PA in cancer survivors. Many studies have investigated behavioral changes using mobile phones in general populations (9), but rarely in cancer survivors. Moreover, this study is the first to be performed for the purpose of decreasing mental distress using mobile devices in cancer survivors. Studies using mobile tools have mainly focused on behavior changes or diet in general populations, among whom mental distress is relatively not a bigger issue than in cancer survivors, most of whom experience psychological problems that have huge impacts on them for the rest of their lives (3).

Studies have used mobile tools simply for providing self-monitoring, feedback, or information (11–13). The concept of a mobile community that supports and encourages participants has been used in a few studies. A study that used web- and app-based communities to promote healthy lifestyles showed that these tools significantly decrease body weight, body fat percentage, and waist circumference after 38 weeks of intervention (14). Another study illustrated that “social” apps with group-based collaboration and competition could significantly reduce overall amounts of sedentary lifestyle and increase PA (15). However, although mobile devices have been shown to improve lifestyle and body composition, the actual influence of these small changes on physical health remains questionable. In the present study, a mobile app-based community was used to enhance weekly steps and reduce mental distress in breast cancer survivors. The importance of this finding is that health promotion using mobile devices can have a significant impact on the mental health among breast cancer survivors, with respect to their daily lives.

To develop interventions that can enhance PA in cancer survivors, theory-based research should be conducted (16). Social cognitive theory, for example, includes the important constructs of outcome expectation following a specific behavior (17). Social outcomes are one of the expected outcomes, including social reactions such as approval. Self-evaluative outcomes, meanwhile, include one's own reaction. Another is theory of planned behavior, which suggests that behavior is determined by intention, which in turn is determined by attitude, subjective norm, and perceived behavioral control (18). Subjective norm refers to the perceived social pressure that individuals feel to perform or not perform. In the present study, the mobile community platform seemed to work based on the abovementioned theories to change participants' behavior.

This study had much strength, including its longitudinal tracking involving multiple time points for both walking activity and mental health. We collected enough data points before and after the initiation of community activity. The study was not randomized; as such, while this research did have the advantage of mimicking the real-life choice of selecting whether to enroll in the community or not, causality could not be assumed. Notably, we used statistical approaches, namely, DID and PSM, that allowed us to model the effects of intervention without a natural time trend and a group difference at baseline. DID is a statistical technique used in econometrics and quantitative research that attempts to mimic an experimental research design using observational study data, by studying the differential effects of an intervention on a treatment group vs. a control group in a natural experiment (19). In our PSM, our pseudo-control and community-later groups were extremely well-matched statistically at baseline. However, future research using a randomized control trial is required to confirm whether involvement in the community is causally linked with better mental health and weekly walking activity.

As the number of cancer survivors has increased around the world (20), the importance of cancer survivorship care has been emphasized. Various technologies developed recently have the potential to launch a new chapter in cancer survivorship care. As a part of such efforts, we conducted this prospective study as well as launched a randomized controlled study to develop distress screening algorithms using mobile device-based PA data in breast cancer survivors in the Distress Reduction by Activity Tracking and Activity Enhancement by Mobile Support Group in Oncology study (DRAAGON study, NCT03783481). We expect these studies to provide new tools to enhance the level of the management of cancer survivorship.

The limitations of the present study should be noted. First, all participants were female breast cancer patients. Thus, our findings can only be generalized to this group of patients. Second, this study was performed in a single tertiary hospital in the Republic of Korea. Third, we did not conduct a randomized controlled trial. Therefore, inherent bias may have influenced the results of the study. Finally, the long-term effects of a mobile community on PA and mental distress have not been demonstrated; the follow-up period of this study was relatively short.

In conclusion, the mobile app-based community is an effective tool to increase PA in breast cancer survivors, which may lead to a decrease in mental distress. The potential role of mobile devices in the management of distress among breast cancer survivors should be further investigated in future.

## Data Availability Statement

The datasets generated for this study are available on request to the corresponding author.

## Ethics Statement

The studies involving human participants were reviewed and approved by the institutional review board at Asan Medical Center (2017-0328). The patients/participants provided their written informed consent to participate in this study.

## Author Contributions

IC, JL, YM, and SC designed the study and provided input throughout the study. YP, HC, HP, and ML collected the data. SC, BS, and S-HA provided clinical expertise. MJ, DC, SL, and IC analyzed portions of the data. IC and MJ wrote the manuscript along with contributions from all of the authors. All authors read and approved the final manuscript.

### Conflict of Interest

HC is CEO of Swallaby Co., Ltd., Seoul, Korea. The remaining authors declare that the research was conducted in the absence of any commercial or financial relationships that could be construed as a potential conflict of interest.

## Supplementary Material

Supplementary Figure 1 Comparison of weekly step counts in the mobile community-first group. Boxplot means ±1^*^standard deviation of sample.
