# Supplementary figures and images for: Exercise Promotion and Distress Reduction Using a Mobile App-Based Community in Breast Cancer Survivors
Source: Front Oncol. 2020 Jan 10;9:1505. doi: 10.3389/fonc.2019.01505 (PMC6966488; doi:10.3389/fonc.2019.01505)

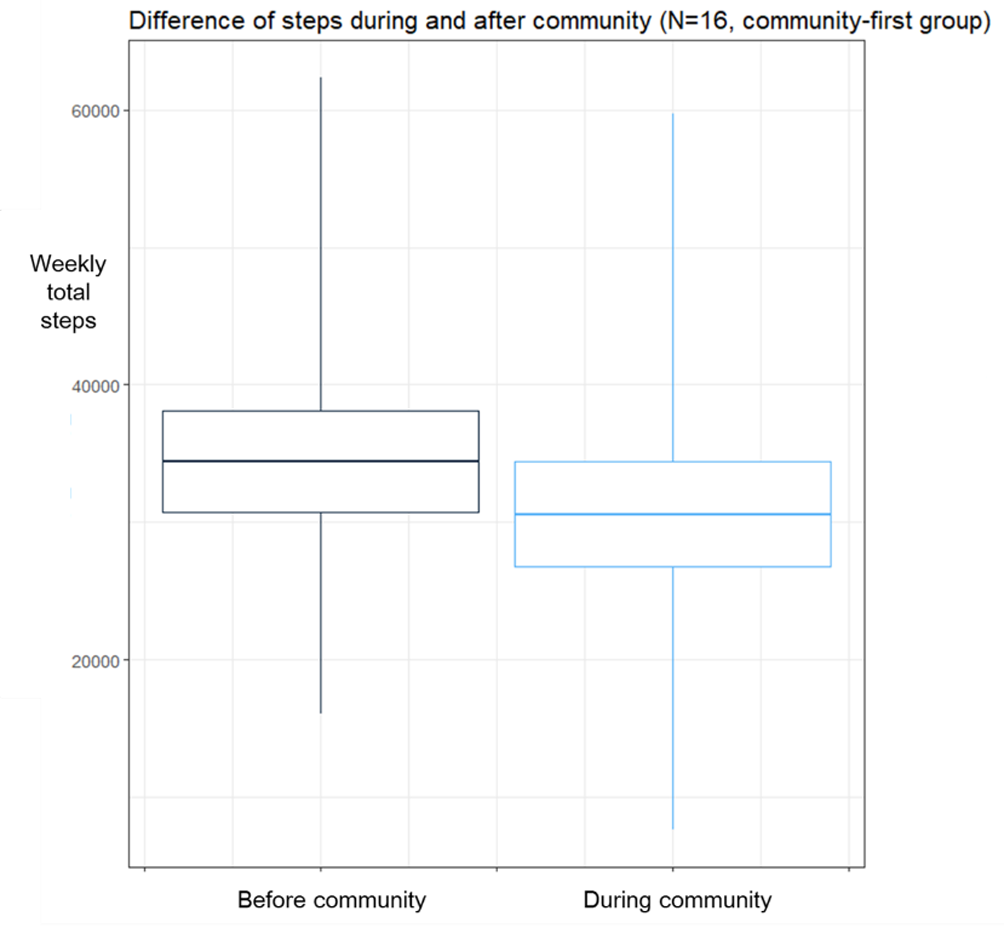

Supplement: Supplementary Figure 1 — Comparison of weekly step counts in the mobile community-first group. Boxplot means ±1*standard deviation of sample. [file Image_1.TIF]
